# Autophagy Modulation Influenced the Spermatogenesis in Rodent Testicular Organoids and Diabetic Condition

**DOI:** 10.34172/apb.46240

**Published:** 2026-06-29

**Authors:** Shadi Atazadeh, Maryam Hasan Sadeh, Farah Farokhi, Shahin Ahmadian, Sepideh Saghati, Fereshteh Valipour, Amir Fattahi, Amin Tamadon, Reza Rahbarghazi, Mahdi Mahdipour

**Affiliations:** ^1^Stem Cell Research Center, Tabriz University of Medical Sciences, Tabriz, Iran; ^2^Department of Biology, Faculty of Science, Urmia University, Urmia, Iran; ^3^Department of Dental Biomaterials, Faculty of Dentistry, Tabriz University of Medical Sciences, Tabriz, Iran; ^4^Department of Reproductive Biology, Faculty of Advanced Medical Sciences, Tabriz University of Medical Sciences, Tabriz, Iran; ^5^Department of Natural Sciences, West Kazakhstan Marat Ospanov Medical University, Aktobe, Kazakhstan; ^6^Stem Cell and Regenerative Medicine Institute (SCARM), Tabriz University of Medical Sciences, Tabriz, Iran; ^7^Department of Applied Cell Sciences, Faculty of Advanced Medical Sciences, Tabriz University of Medical Sciences, Tabriz, Iran

**Keywords:** Alginate/gelatin hydrogel, Testicular organoids, Autophagy, Diabetes mellitus, Spermatogenesis

## Abstract

**Purpose:**

Here, the effect of autophagy activity was investigated in rat testicular organoids embedded in alginate/gelatin (Alg/Gel) hydrogel. Additionally, we monitored the spermatogenesis potential in diabetic mice following the administration of metformin (Met) and/or hydroxychloroquine (HCQ).

**Methods:**

Alg/Gel hydrogel was fabricated, and its physicochemical properties were studied. The survival rate of freshly isolated rat whole testicular tissue cells being plated on the Alg/Gel hydrogel surface was monitored after 7 days. Rat testicular organoids were embedded in Alg/Gel hydrogel and exposed to 15 µM HCQ and/or 5 µM Met for 7 days. Histological examination was conducted to assess OCT3/4+ progenitors and maturing sperm cells. Using western blotting, the levels of autophagic markers Beclin-1, LC3, and P62 were evaluated. Diabetic mice received 120 mg/kg body weight Met and/or 70 mg/kg bodyweight HCQ for 4 weeks, followed by histological examinations and sperm analysis.

**Results:**

The fabricated Alg/Gel hydrogel demonstrated suitable physicochemical properties, which correlated with an increased survival rate of whole testicular tissue cells compared to the control group (*P*<0.05). Data indicated a promotion of autophagy (Beclin-1↑, LC3-II/-I ratio↑, P62↓) and an inhibition of autophagy (LC3↓) in testicular organoids after treatment with Met and HCQ, respectively, compared to the control group (*P*<0.05). These changes were accompanied by an induction of SOD activity in Met-treated testicular organoids (*P*<0.05). In the presence of Met and HCQ, OCT3/4+ progenitors were reduced in rat organoids compared to non-treated control organoids (*P*<0.05). Administration of Met, but not HCQ, in diabetic mice was able to restore some sperm parameters, such as motility, and reduce abnormal morphologies compared to the matched diabetic mice (*P*<0.05).

**Conclusion:**

Regulation of autophagy can influence spermatogenesis capacity, affecting seminiferous tubule progenitors, maturation, and sperm parameters. In studies of rat testicular organoids and diabetic mice, these findings suggest that the autophagy response is a promising signaling pathway target in subfertile or infertile males.

## Introduction

 Infertility is a global clinical issue with the inability to conceive after 12 months of unprotected intercourse.^1^ Infertility can affect both males and females of reproductive age with socioeconomic burdens.^2^ In males, genetic makeup, congenital disorders, senile changes, metabolic disorders, medical, physical, and environmental factors, and other pathological conditions can lead to infertility related to impaired or weak ejaculation capacity, sperm morphology, motility, and number.^3,4^ Both reduced sperm count (oligozoospermia), and absence of sperm in semen (azoospermia) can increase the possibility of infertility in males.^5^ Nowadays, several ART strategies such as *in vitro* fertilization, testicular sperm extraction, intracytoplasmic sperm injection, etc., along with hormone therapy (i.e. estrogen receptor modulators) and antioxidants are helpful in the restoration of the male reproduction system to have a child without satisfactory outcomes.^6,7^ Along with these statements, development and finding therapeutic strategies are at the center of attention.^8^

 The phenomenon of spermatogenesis is an intricate mechanism with the close collaboration of different cell lineages and the balance of sex-related hormones.^9^ Besides, it was suggested that maintaining the balance between degradation, and bioenergetic supply has profound impacts on spermatogenesis and steroidogenesis.^10^ In this regard, previous studies have unveiled the essential role of autophagic response in the regulation of various cell bioactivities such as the elimination of injured organelles, and misfolded proteins from the host cells. These features can influence cell homeostasis, dynamic growth activity, maturation, and differentiation.^11^ The activation of autophagy occurs in response to various insulting, and stressful conditions and exposure of host cells to harsh microenvironments, leading to the activation of diverse downstream effectors, namely autophagy-related genes.^12^

 Under diabetic conditions, the increase of glucose contents and byproducts can directly affect the testicular microenvironment and cell function such as Leydig, and Sertoli cells. In response to hyperglycemic conditions, the stimulation of apoptotic changes in Leydig cells can increase the possibility of structural changes in the testes.^13^ Likewise, certain microanatomical structures such as blood-testis barrier integrity are lost following the activation of different molecular signaling pathways and accumulation of excessive free oxidant radicals.^14^ It has been shown that adaptive autophagy can reduce the detrimental effects of several pathological conditions on the reproduction system.^15^ However, the lack of appropriate autophagic response, or an abnormal and excessive autophagy stimulation, i.e., under diabetic conditions, can distort the homeostatic properties of testicular tissue cell functions.^16^ Thus, the restoration of adaptive autophagy function is thought to alleviate the pathological conditions, having profound impacts on fertility beyond spermatogenesis.

 In recent decades, the advent of 3D culture techniques has helped biologists to recapitulate *in vivo*-like microenvironments at the laboratory scale.^17^ Using different 3D culture strategies such as cell aggregation, and natural and synthetic biomaterials it is possible to precisely monitor underlying molecular mechanisms in regenerative medicine area. ^17,18^ Despite these advantages in the monitoring critical role of autophagy in the process of spermatogenesis, few reports exist related to the modulation of autophagic response in 3D cultured testicular tissue units in an *in vitro* setting. Therefore, this study aimed to explore the effects of autophagy inhibition/activation on the promotion of spermatogenesis using 3D alginate-gelatin (Alg/Gel)-based hydrogels in *in vitro* conditions (**graphical abstract**). The possible effects of autophagy response, either in inhibitory or stimulatory states, were also assessed under diabetic mice.

## Materials and Methods

###  Alg /Gel Hydrogel Synthesis Protocol 

 Here, in this study, Alg/Gel hydrogel was fabricated using an ionic cross-linker CaCl_2 _according to previously published data.^19^ In short, sodium Alg powder (I-1G, high content of guluronic acid, and MW: 70 kDa; Kimica; Japan) was sterilized using 70% EtOH solution and dissolved in calcium-free Krebs Ringer HEPES-buffered saline with adjusted pH of 7.4. Gel (CAS 9000-70-8; Sigma-Aldrich) was sterilized using chloroform at 4°C. The mixture of Alg and Gel was prepared by adding Gel to the Alg solution to create a hydrogel with concentrations of 1% w/v for both Alg and Gel. The procedure continued with the addition of 0.2 M CaCl_2_ to facilitate the cross-linking of Alg and Gel.

###  Physicochemical Properties 

####  Fourier-Transform Infrared Spectroscopy (FTIR) 

 ATR-FTIR spectroscopy was used to monitor the chemical structure of Alg/Gel hydrogel and the efficiency of the synthesis protocol based on the standard protocols.^20^ In brief, Alg/Gel hydrogel (n = 3) was frozen at -20˚C and immediately lyophilized. The attenuated total reflectance technique of the ATR-FTIR spectrum of Alg/Gel hydrogel was analyzed by a Varian 610 spectrometer, with a DTGS detector and a diamond crystal.

###  Degradation Rate

 This assay evaluates the degradation and destruction of hydrogels in the target aqueous phases.^21^ Alg/Gel hydrogels were synthesized in cylindrical molds (1 x 1 × 1 cm), and placed in a normal saline solution at 37°C for 11 days. Samples were collected on days 0, 4, 7, and 11, and their weights were recorded (W_0_). The samples were then stored at -20°C and later lyophilized. After lyophilization, the weights were measured again (W_1_) (n = 4). The degree of weight loss was calculated using the following equation with four replicates.


(1) $$Weight\;loss\;\%=\frac{\left(W1-W0\right)}{W0}\times 100$$

###  Swelling Ratio 

 The swelling capacity of Alg/Gel hydrogel was monitored after being incubated in phosphate-buffered saline (PBS) at 37˚C. On various time intervals including, 1, 6, 12, 24, 48, and 72 hours, samples were taken and weighed after removing the surface water using filter papers (n = 4).^22^ The swelling ratio was calculated using the below equation, where W_0_ and W_1_ are the initial and swelled weights of the hydrogels respectively at different time points. This assay was performed in triplicate.


(2) $$Swelling\;ratio\;\%=\frac{\left(W1-W0\right)}{W0}\times 100$$

###  Mechanical Properties 

 An extensometer instrument was applied to evaluate the mechanical pressure tolerance of fabricated Alg/Gel hydrogel. Cylindrical scaffold samples (1 x 1 × 1 cm) were synthesized and subjected to a compression rate of 2 mm per minute (n = 3). The second Young’s modulus was determined as the slope of a linear fit to the stress-strain curve. The relationship between stress, strain, and Young’s modulus was analyzed using the following formula: *Stress = E × Strain*.

###  SEM Imaging

 Hydrogels were fixed in 2.5% glutaraldehyde solution and subjected to dehydration using ascending EtOH series. Both cell-free and cell-loaded samples were gold-sputtered and the microstructure features were imaged using the FE-SEM system (Mira-3 FEG SEM microscope, Tescan Co., Czech) under an acceleration voltage of 15kV.

###  Animals and Ethics

 The *in vitro* and *in vivo* phases of the present study were approved by the local ethics committee of Tabriz University of Medical Sciences (IR.TBZMED.REC.1401.280) and animals were treated according to the “Principles for the Care and Use of Laboratory Animals” (NIH, 1996). Animals were kept inside standard cages and under standard environmental conditions with free access to tap water and chewing pellets.

###  MTT Assay

 To evaluate the effects of a fabricated Alg/Gel hydrogel on whole testis cells, an MTT assay was conducted. For this purpose, male Wistar rats, ranging between 20-250 g, were humanly euthanized using an overdose of Ketamine and Xylazine. Under sterile conditions, testes were sampled and transferred to the Cell Culture Lab of the Stem Cell Research Center affiliated with Tabriz University of Medical Sciences. Tissues were chopped into small pieces and then subjected to enzymatic digestion using 0.1% type I collagenase (9001-12-1, Sigma-Aldrich) for 10-20 minutes. The enzyme activity was neutralized using fetal bovine serum (FBS; Gibco) solution, and the suspended cells were passed through 200 µm pore-sized meshes. The cells were collected, counted using a hemacytometer slide, and mixed with Alg/Gel solution (2 × 10^6^ per 1 mL of hydrogel). The mixture was incubated with 200 mM CaCl_2_ for solidification of Alg/Gel mixture. Approximately 50 µL of hydrogel containing 1 × 10^4^ cells were transferred onto each well of a 96-well plate and maintained for 7 days under standard culture conditions (37˚C, 95% relative humidity, and 5% CO_2_). After the completion of the incubation period, the supernatants were discarded and replaced with 100 µL MTT solution (2 mg/mL) and kept at 37˚C for 4 hours. The procedure continued with the addition of a DMSO solution, and the OD values were measured at 590 nm using a microplate reader (Model: Elx808; BioTek). In the control group, isolated cells were plated on a hydrogel-free plastic surface for comparison with a similar initial cell density. The data were expressed as a % of the control group. The procedure was performed in 15 replicates per group.

###  Testicular Organoid Culture

 In this study, we developed a 3D testicular organoid culture system using Alg/Gel hydrogel. The rat testes were cut into 2 mm slices. After that, 400 µl Alg/Gel hydrogel was poured onto each well of 24-well plates. A single testicular tissue fragment was placed in each well and the procedure was followed by overlaying an additional 400 µl Alg/Gel hydrogel. The mixture was polymerized using a CaCl_2_ solution. To exclude the toxicity of excessive calcium ions, each well was washed three times using PBS. The fragments were incubated with DMEM/HG culture medium containing 10% FBS and 1% Pen-Strep solution under standard conditions for 7 days. To promote active dynamic growth and induction of spermatogenesis cultured testicular organoids, 10 µM testosterone (Aburaihan Co.), 5 µM retinoic acid (Cas No: 302-79-4, Sigma-Aldrich), and 100 ng/mL rFSH (CinnaGen; Tehran; Iran) were added into the culture medium.^23^ The culture medium was replenished every 3-4 days. This procedure was performed in triplicates per group.

###  Modulation of Autophagy Response 

 To this end, testicular organoids within the Alg/Gel hydrogel were exposed to 5 µM metformin (Met; Osvah Pharmaceutical Co.; Tehran; Iran), or 15 µM hydroxychloroquine (HCQ; Cat No: H0915, Sigma-Aldrich) to modulate the autophagy response.^24-26^ The culture medium was renewed every 3-4 days and organoids were kept under standard culture conditions for 7 days.

###  Western Blotting

 After 7-day culture time, incorporated testicular organoids were removed from supporting Alg/Gel hydrogel after 5 minutes using 0.1 M sodium citrate followed by washing steps (3X).^27^ Organoid masses were lysed using protein lysis buffer [0.1% SDS, 150 mM NaCl, 50 mM Tris HCl, 1% NP-40, 2 mM EDTA] overnight at 4°C. The lysates were centrifuged at 11,000 rpm for 20 minutes at 4°C. Protein contents were measured using BCA assay, and 10 µg protein from each group was electrophoresed on 10% SDS-PAGE gel. After transferring onto the PVDF membranes, a panel antibody including anti-human Beclin-1 (Cat no: sc-48341; Santa Cruz Biotechnology, Inc.), LC3 (Cat no: Sc-398822; Santa Cruz Biotechnology, Inc.), and P62 (Cat no: Sc-10117; Santa Cruz Biotechnology) was used to monitor autophagy effectors. Membranes were incubated with primary antibodies overnight at 4°C. After three PBS washes, HRP-tagged secondary antibodies (Cat no: sc-2357, Santa Cruz Biotechnology, Inc.) were used for labeling of immunoreactive bands on X-ray films after the addition of ECL solution. Using ImageJ software (ver.1.4; NIH), the density of each immunoreactive band was calculated. The values were expressed as relative protein levels compared to housekeeping protein β-actin (Cat no: sc-517582; Santa Cruz Biotechnology, Inc.). This procedure was performed in triplicate for each protein tested.

###  H & E Staining

 The tissue structure and further microstructure changes were monitored using H & E staining. On day 7, organoid-laden Alg/Gel hydrogels were gently removed and fixed using a 10% buffered formalin solution. After passing dehydration, and rehydration steps, the paraffin-embedded samples were cut into 5 µm-thick slices and stained using H & E solution. Three sections per sample were prepared, and the entire section was evaluated for histopathological assessments. To analyze the cellular population within the seminiferous tubules, over 20 high-power fields were randomly selected, and the cells in different developmental stages were noted. WHO guidelines were implemented to annotate cells at various stages within the seminiferous tubules.

###  Immunofluorescence (IF) Staining 

 To monitor the influencing effects of autophagy on the density of tissue progenitor cells in testicular organoids, IF staining was performed in terms of Oct3/4^+^ cells (n = 3 per group).^28^ Organoid-laden Alg/Gel hydrogels were incubated with OCT compound, and snap-frozen at -20°C. Using the cryosection technique, 5 μm-thick slices were prepared. In the next step, samples were allowed to be thawed and washed three times with PBS. Then, samples were treated with 0.5% Triton X-100 for 5 minutes to permeabilize the cell membrane. The procedure was continued with the blocking of samples using 1% FBS for 5-10 minutes and anti-Oct3/4 antibody (Dilution: 1: 500; Cat no: sc-5279; Santa Cruz Biotechnology, Inc.) overnight at 4°C. Afterward, samples were washed three times with PBST (3 × 10 minutes) and incubated with FITC-conjugated secondary antibody (Cat no: sc-2010; Santa Cruz Biotechnology, Inc.) for 1 hour at RT. After three PBST washes, DAPI (1 µg/ml; Sigma-Aldrich) was used for nuclear staining. Samples were imaged using a fluorescence microscope (Model: BX41; Olympus; Japan).

###  Oxidative Stress Status

 The oxidative status of oxidative status in testicular organoid-loaded Alg/Gel hydrogel was also studied after treatment with 5 µM Met or 15 µM HCQ. The protein lysates were used for measuring glutathione peroxidase (GPx; RANDOX Laboratories), superoxide dismutase (SOD; RANDOX Laboratories Ltd), and total antioxidant capacity (TAC; RANDOX Laboratories) levels according to the manufacturer’s instruction. This procedure was performed in triplicates per group.

###  Induction of Diabetes Mellitus (DM) 

 Fifteen BALB/c male mice, ranging from 25-30 g, were randomly selected for induction of long-lasting DM using streptozotocin (STZ; Cat no: S0130; Sigma Aldrich).^29^ The recommended dose was dissolved in solution containing same volume of sodium citrate (2.94 g/100 ml; Sigma-Aldrich) and citric acid (2.1 g/100 ml water; Sigma-Aldrich) solution (pH = 4.5). STZ at a dose of 50 mg/kg body weight was administrated in three separate injections via an intraperitoneal route with an average interval of 2 days. Four days after the last injection, the systemic levels of glucose were monitored using a glucometer (Model: TD-4277; Aktivmed GlucoCheck XL). Levels of more than 250 mg/dL were conceived as diabetic conditions.

###  Effect of Met and HCQ in Testicular Tissue of Diabetic Mice

 Mice were randomly divided into three groups (each in 5) as follows; non-treated Control, DM, DM + Met, and DM + HCQ groups. In DM + Met mice, 120 mg/kg of body weight Met was injected via i.p. once per week for 4 weeks into the DM mice. This protocol was implemented to investigate the effects of sustained, long-term modulation rather than acute daily fluctuations. In the DM + HCQ group, the DM mice received 70 mg/kg body weight HCQ once a week for 4 weeks. Distilled water (DW) was used as a vehicle for all injections. In the control mice, DW alone was used as the control vehicle. Additionally, animals were monitored daily for clinical signs of toxicity and weight loss, and no adverse effects were observed. One week after the last injection, the mice were euthanized using an overdose of ketamine, and xylazine, and epididymis was used for the analysis of sperm parameters. Testes were fixed inside a 10% buffered formalin solution and subjected to H & E staining as above-mentioned. For histological examination, the number of spermatogonial cells, spermatocytes, and spermatids were counted in each seminiferous tubule in slides. The spermogram analysis was conducted in a blinded manner according to WHO guidelines, using epididymal semen to assess parameters such as total sperm count, abnormalities, and motility rates (%) (n = 5).

###  Statistical Analysis

 Data (Mean ± SD) were analyzed using GraphPad Prism software (ver. 8.4.3.). Student t-tests and ANOVA Tukey’s post hoc test were applied to find any statistical differences between the experimental groups. *P* < 0.05 was used to describe the existence of significant differences between the groups.

## Results and Discussion

###  Alg /Gel Hydrogel Physicochemical properties

####  FTIR 

 Using FT-IR spectra of Gel, Alg, and Alg/Gel hydrogel, the chemical structures were monitored (Figure 1A). Based on data, Alg alone exhibits two characteristic absorption bands at 1617 cm^−1^ and 1418 cm^−1^ and is attributed to the asymmetric and symmetric stretching vibration of the -COO group, respectively. An adsorption band at 3446 cm^−1^ is related to the stretching vibration of the hydroxyl functional group and a stretching vibration absorption peak of the -CH_2_ group at 2926 cm^-1^.^30^ The characteristic absorption bands of gelatin at 1648 cm^−1^, 1540 cm^−1^, and 1238 cm^−1^ were attributed to amide I (C = O and C-N stretching), amide II and amide III (mainly N-H bending, and C-N stretching vibration), respectively. Finally, the wide absorption bond around 3421 cm^−1^ was due to the N-H stretching vibration.^31^ From the FT-IR spectra of Alg/Gel hydrogel, it was indicated that the characteristic strong absorption band at 1648 cm^−1^ (amide I) of Gel shifted to a lower wave number at 1637 cm^−1^ and indicated the interaction between Gel amino and Alg carboxylic groups (Figure 1A). Moreover, the slight shift of the peak of amide I from 1647 cm^-1^ to 1637 cm^−1^ in the Alg/Gel composite confirms the fact that negative groups of Alg can interact with positively charged groups of Gel.^32^ The peak at 1261 cm^−1^ (amide III) indicates the presence of Gel in the final composite. At the same time, the absorption –NH band of Gel at about 3421 cm^−1^ and –OH band of Alg at about 3421 cm^−1^ broadened and shifted to a lower wave number at 3417 cm^−1^, suggesting an increase in the hydrogen bonding (Figure 1A).^32,33^ All those changes show strong evidence of the intermolecular interactions between –OH groups of Alg and –NH groups of Gel molecules.

**Figure 1:**
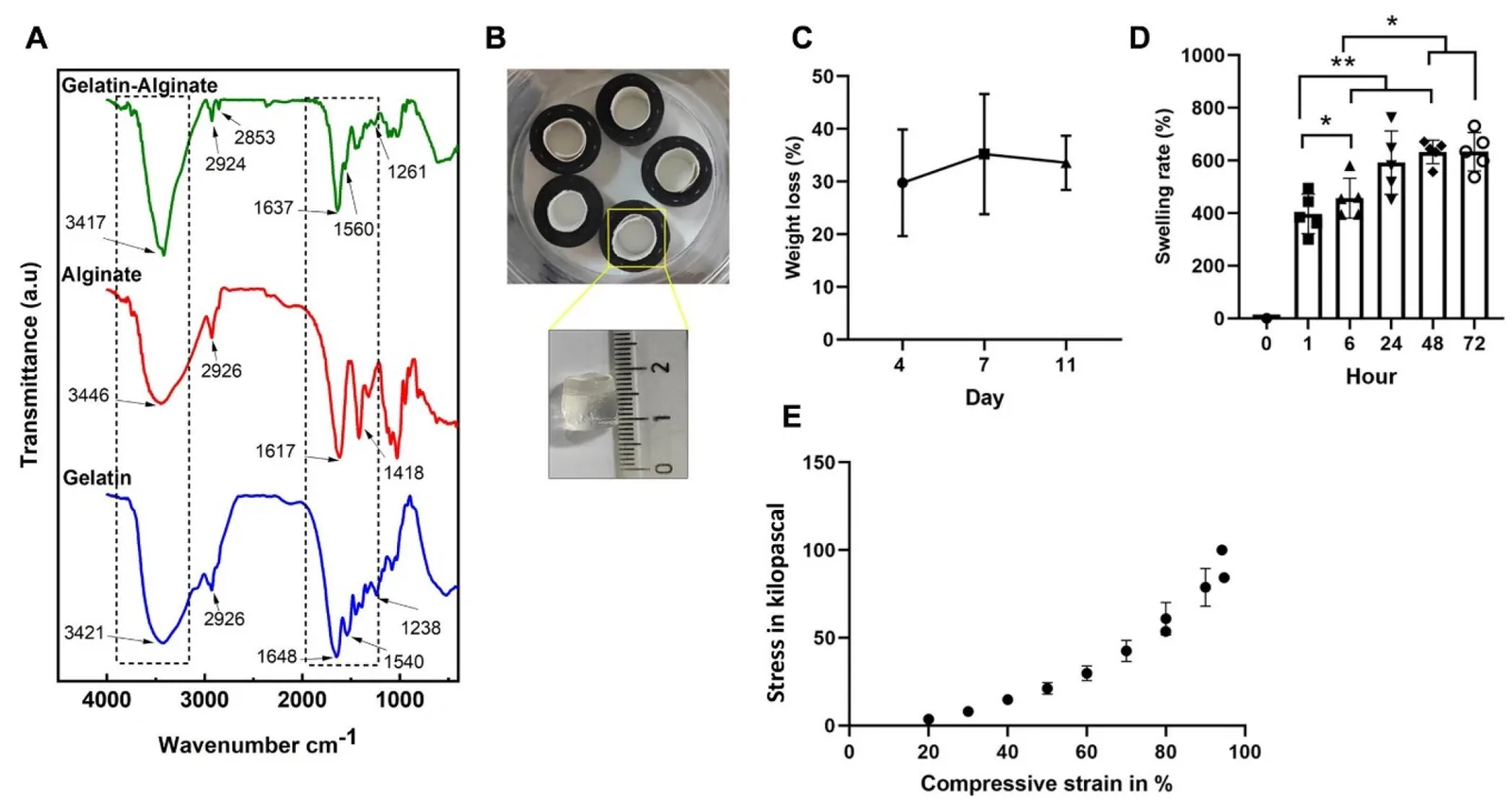
FTIR analysis of Alg/Gel composite after ionic cross-linking using CaCl_2_ (**A**; n = 4). The suspension of Alg/Gel hydrogel was cast using cylindric models followed by the addition of 200 mM CaCl_2_ for ionic cross-linking (**B**). Monitoring degradation rate (**C**; n = 4), Swelling ratio (**D**; n = 4), and mechanical properties (**E**; n = 3). All quantified data are presented as mean ± SD

###  Biodegradation, Swelling, Mechanical Properties 

 In this study, Alg/Gel hydrogel masses were fabricated using cylindric molds for assessing physicochemical properties (Figure 1B). Biodegradation analysis revealed 30% weight loss in the first 4 days, and these values reached 35% on day 7 (Figure 1C). Based on our findings, the incubation of Alg/Gel hydrogel in the aqueous phase for 11 days can contribute to 33% weight loss (Figure 1C). Taken together, it can be told that the existence of degradation in fabricated Alg/Gel hydrogels makes them responsive to the biological fluids, and replacing with the natural matrix over time.

 Data confirmed the swelling capacity and ability to absorb water over 72 hours (Figure 1D). We noted that Alg/Gel hydrogels show an upward trend in weight gain after 1, 6, 24, and 48 hours. Also, the water absorption capacity of Alg/Gel substrates was relatively constant after 48 hours in which no statistically significant differences were achieved in terms of swelling ratio at time points 48 and 72 hours (*P* > 0.05; Figure 1D). According to the obtained data, the fabricated Alg/Gel hydrogel scaffold exhibited relatively appropriate resistance to mechanical pressure (Figure 1E). The samples measured in three repetitions were broken at an average strain of about 89.70%. Also, the ultimate compressive strength (UCS) was evaluated as 45.97 Kilopascal on average (Figure 1E).

###  SEM Imaging 

 SEM images the existence of pores within the Alg/Gel hydrogel (Figure 2A-B). Based on our estimation, the average size of pores reached 36.28 ± 13.61 µm (Figure 2B). These data show the existence of a porous structure within the fabricated Alg/Gel hydrogel. Further analysis indicated the attached cells inside the polymeric network with the acquisition of flattened morphologies. These features support the notion that Alg/Gel hydrogel provides a suitable platform for attachment and suitable morphology of incorporated cells (Figure 2C).

**Figure 2:**
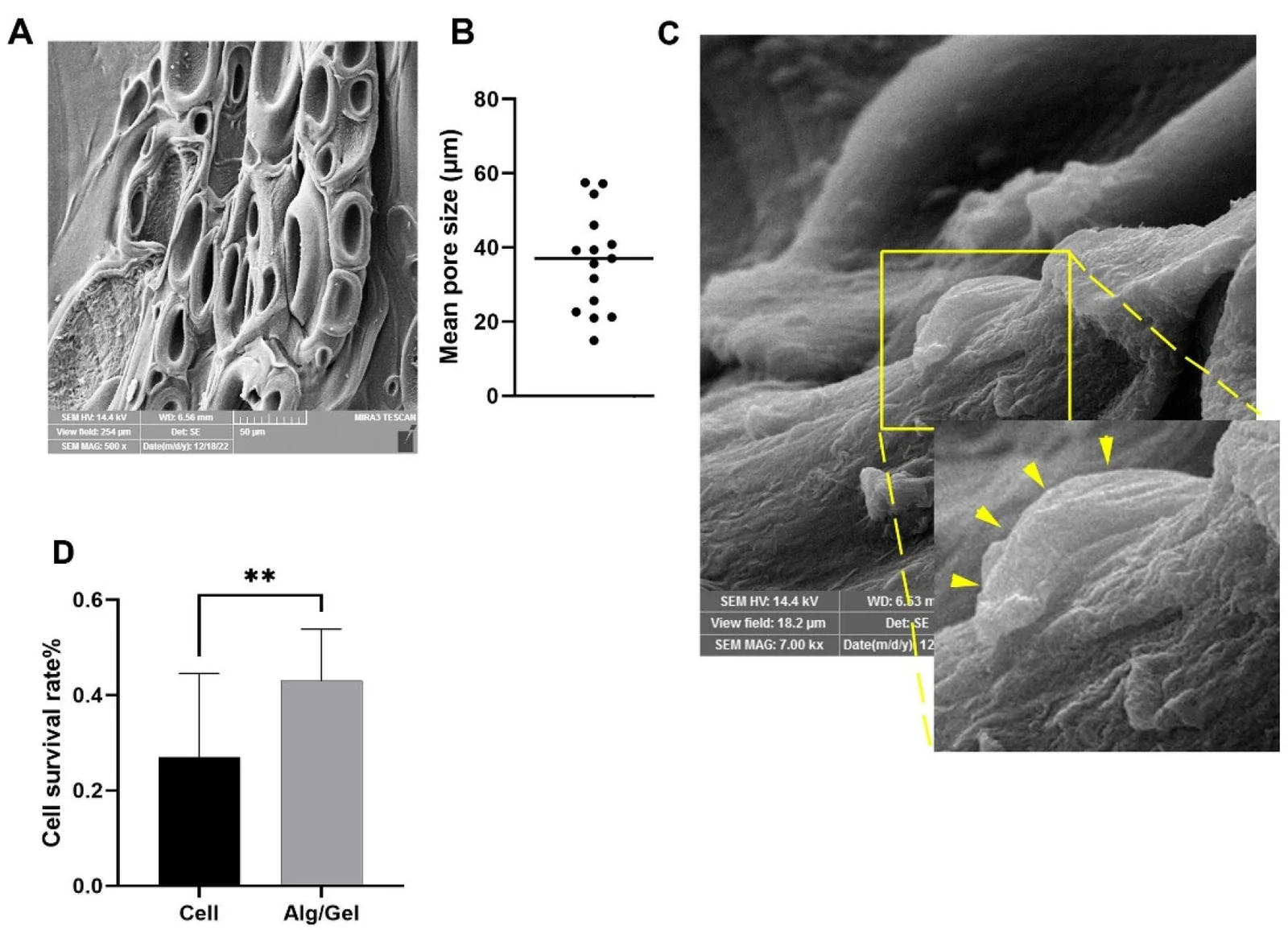
SEM analysis of Alg/Gel hydrogel porosity (**A**), and cell adhesion (**B**). The yellow arrowheads indicate flattened cell mass adhered tightly to the Alg/Gel surface after 7 days of culture in *in vitro* conditions (**C**). Monitoring the survival rate of whole testicular tissue cells after 7 days plated on Alg/Gel hydrogel compared to control cells cultured on a plastic surface (**D**; n = 15). Student t-test. **P< 0.01. All quantified data are presented as mean ± SD

###  Alg/Gel Substrate Induced Survival Rate of Testis Cells 

 Conventional MTT assay indicated that a 7-day culture of freshly isolated testis cells led to an enhanced survival rate when compared to the control group (*P* < 0.01; Figure 2D). These features show that Alg/Gel hydrogel provides a suitable microenvironment for the induction of viability of testis cells.

###  Autophagy Modulation in Testicular Organoids

 The autophagic response was monitored in Met, and HCQ-teared testicular organoid-loaded Alg/Gel hydrogels after 7 days *in vitro* (Figure 3A-B). Data indicated the statically significant increase of Beclin-1, LC3-II/-I ratio coincided with a reduction of P62 in Met-treated groups compared to non-treated control, and HCQ groups (*P* < 0.05). These data indicate that Met can stimulate the autophagic response in the early steps (Beclin-1↑), while the increase of LC3-II/-I ratio and total LC3 levels are indicative of enhanced autophagy flux. The reduction of P62 confirms the completion of autophagic response (Figure 3A-B). On the contrary, the addition of HCQ can blunt the autophagy signaling pathway in which an inverse pattern was evident as compared to the Met-treated group (*P* < 0.05; Figure 3A-B). Compared to the control group, protein levels of Beclin-1, and P62 except for total LC3 content remained unchanged in the HCQ-treated group. Thus, we noted that HCQ can blunt the autophagic response in testicular organoid-loaded Alg/Gel hydrogels via the regulation of autophagic flux. Taken together, data from western blotting confirmed the influencing impacts of Met and HCQ on the autophagy signaling pathway in the 3D cultured testicular tissue fragments after 7 days *in vitro*.

**Figure 3:**
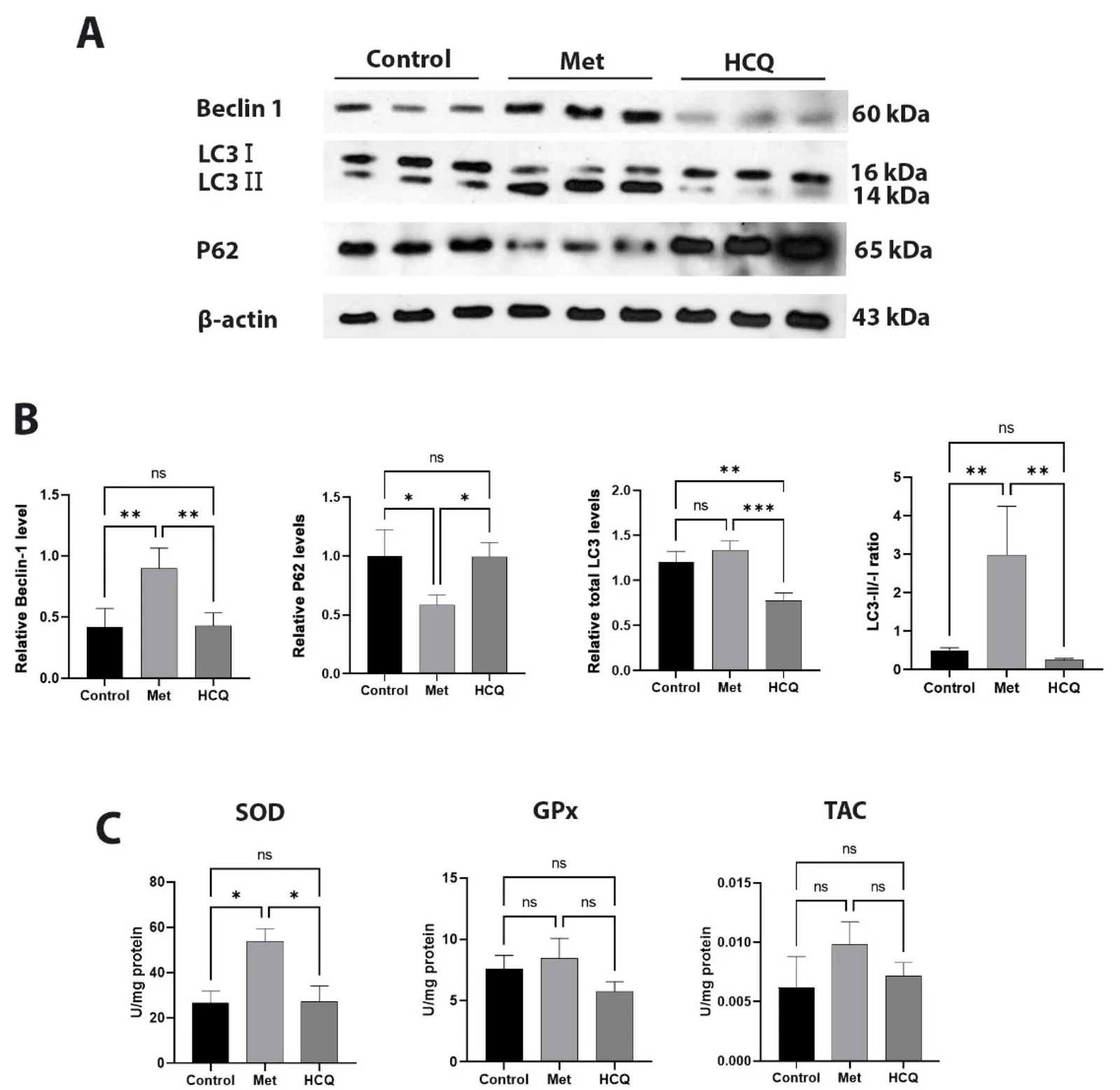
Monitoring the autophagy-related protein levels using western blotting in rat testicular organoids encapsulated inside Alg/Gel hydrogel in response to 5 µM Met and/or 15 µM HCQ after 7 days (**A** and **B**; n = 3). Each column represents the average (mean ± SD) of three biological replicates normalized with β-actin as a loading control (**B**). Data indicated the stimulation and inhibition of autophagy response via the alteration of Beclin-1, total LC3 levels, LC3-II/-I ratio, and P62 in organoid mass compared to the control organoids. The antioxidant capacity of rat testicular organoids was also assessed in terms of SOD, GPx, and TAC after 7 days in Met- and HCQ-treated groups (**C**; n = 3). Data confirmed an activated SOD status and a slight increase in GPx function and TAC levels in the presence of Met but not HCQ. One-way ANOVA with Tukey post hoc analysis. *P< 0.05; **P< 0.01; and ***P< 0.001. All quantified data are presented as mean ± SD

###  Antioxidant Properties were Enhanced in the Met-Treated Testicular Organoids

 Data revealed an increased SOD activity in the presence of Met compared to the control and HCQ-treated groups (*P* < 0.05; Figure 3C). Non-significant differences were found in terms of GPx, and TAC between the groups. Even though, treatment of encapsulated organoids with Met and/or HCQ did not alter the activity of GPx, and TAC in comparison with the non-treated organoids (*P* > 0.05; Figure 3C). Therefore, one can hypothesize that the stimulation of autophagy response can recruit the activity of an early-stage antioxidant enzyme SOD in encapsulated testicular organoids within the Alg/Gel hydrogel after 7 days.

###  Autophagy Modulation Altered the Maturation of Sperm Cells in Testicular Organoids 

 H & E staining was used to follow up any histological changes in testicular organoids in the presence of Met, and/or HCQ after 7 days within the Alg/Gel hydrogel (Figure 4A-B). In the non-treated control group, sperm cells at different developmental stages, both spermatocytes and spermatids, were detected. Bright-field images showed that the number of nucleated cells was reduced in the Met-treated organoids while these changes were less in the HCQ group (Figure 4A). Along with these changes, sperm cell counting revealed the reduction of maturing cells like spermatids and spermatocytes in the lumen of seminiferous tubules in the Met-treated organoids compared to the control and HCQ groups (*P* < 0.001; Figure 4B). Notably, autophagy inhibition using HCQ did not yield statistically significant differences in terms of maturing sperm cells compared to the control organoids (*P* > 0.05).

**Figure 4:**
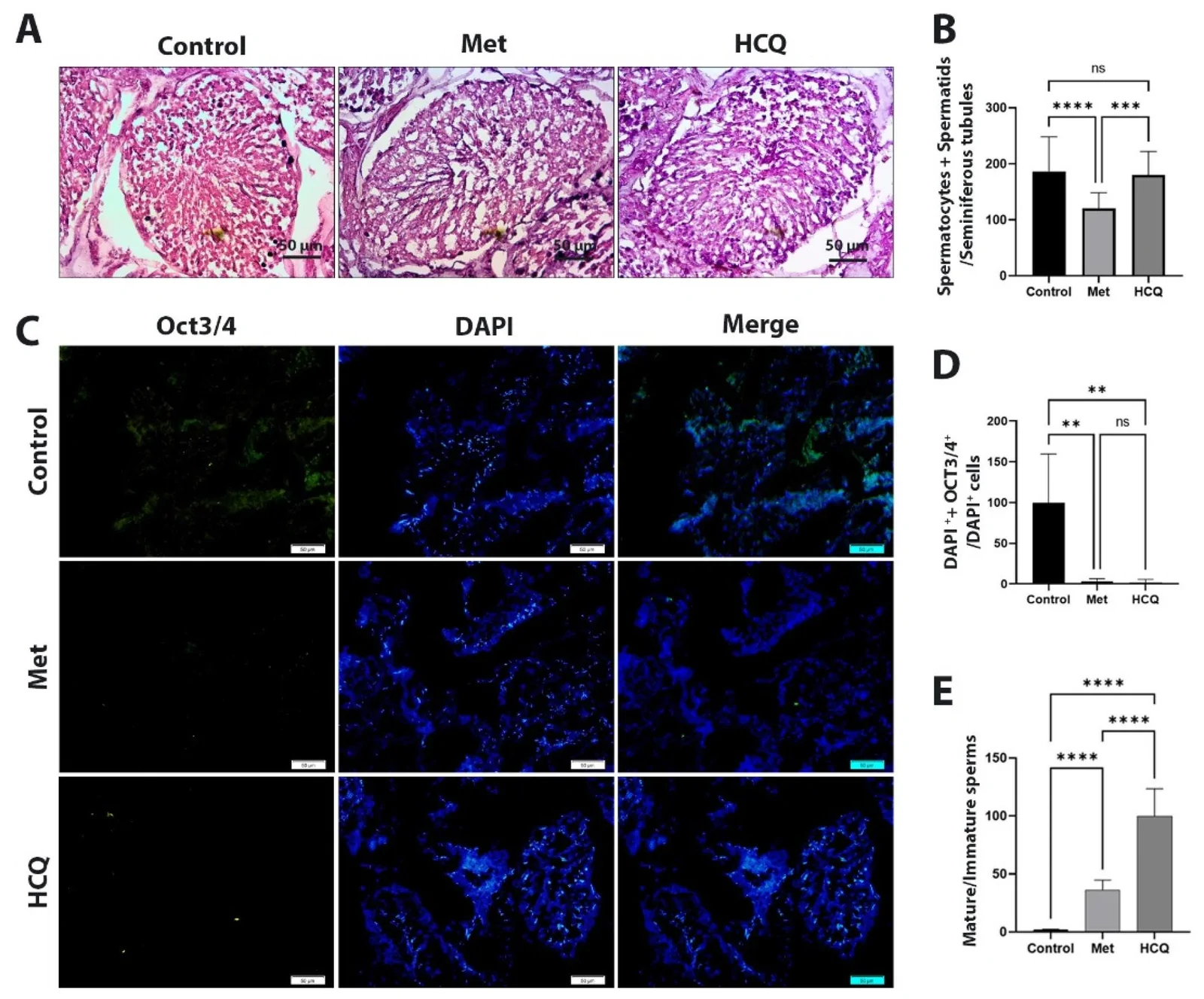
Histological examination (H & E staining) of rat testicular organoids within the Alg/Gel hydrogel after treatment with Met, and HCQ (**A-B**; n = 20). Data indicated the alteration of immature sperm cells per seminiferous tubules within the organoids after 7 days related to non-treated organoids. Monitoring the density of OCT3/4^+^ progenitors inside organoids seminiferous tubules in Met-, and HCQ-treated organoids (**C**, **D**, and **E**; n = 4). Data indicated the reduction of OCT3/4^+^ progenitors and an increase of maturing sperm cells in treated organoids. Scale bar = 50 µm. One-way ANOVA with Tukey post hoc analysis. **P< 0.01; ***P< 0.001; and ****P< 0.0001. All quantified data are presented as mean ± SD

 In parallel with H & E staining, the density of OCT3/4^+^ spermatogonial stem cells per seminiferous tubules was also determined using IF staining (Figure 4C-E). Based on the present data, the number of OCT3/4^+^ spermatogonial stem cells was significantly declined in Met-, and HCQ-treated organoids compared to the control group (*P* < 0.01; Figure 4C-D). The OCT3/4^+^/OCT3/4 - ratio was also calculated to predict the maturation capacity of sperm cells in the presence of Met, and/or HCQ (Figure 4E). The results indicated a significant increase in the ratio of mature to immature cells (OCT3/4 - /OCT3/4^+^↑) ratio in the Met and HCQ groups compared to control organoids (*P* < 0.0001). Besides, the incubation of testicular organoids with HCQ had more significant effects on increased sperm cell maturation (OCT3/4 - /OCT3/4^+^↑) related to the Met-treated group (*P* < 0.0001; Figure 4E). These data indicate that modulating autophagy can influence the regenerative potential of rat testes by regulating the early signs of differentiation in sperm cells.

###  Autophagy Can Alter Sperm Cell Number and Dynamic Growth in Diabetic Mice

 Our findings revealed that both Met and HCQ treatments have suppressed the number of spermatogonial, spermatocyte, and spermatid cells within the seminiferous tubules (Figure 5A-B). Despite the existence of this pattern, non-significant differences were achieved in some sperm cell subsets. Besides, DM can significantly decrease sperm cells in all the developmental stages with a notable decline in the spermatogonial and spermatid cells (*P* < 0001; Figure 5A-B). The administration of Met and HCQ for 4 weeks ameliorated the diabetic condition in terms of sperm cell counts, however, these changes were not statistically significant as compared to the diabetic state (*P* > 0.05; Figure 5A-B).

**Figure 5:**
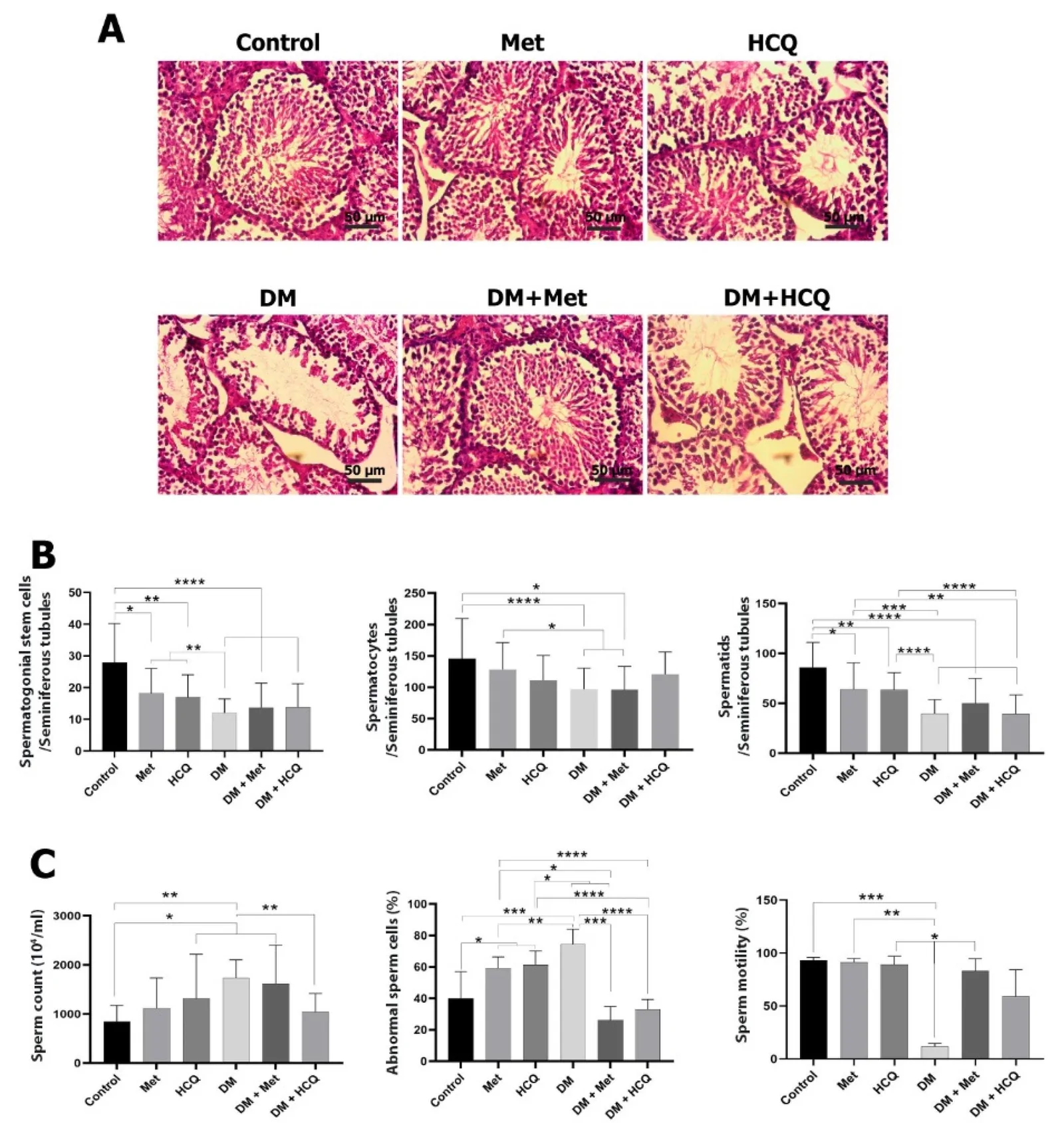
Monitoring histological changes in murine testicular tissue under diabetic conditions after receiving Met and/or HCQ (**A-B**). Spermogram analysis of semen samples from different groups (**C**; n = 5). Data indicated the improvement of some sperm parameters in diabetic mice after administration of Met, but not HCQ after 4 weeks. Scale bar = 50 µm. One-way ANOVA with Tukey post hoc analysis. *P < 0.05; **P < 0.01; ***P < 0.001; and ****P < 0.0001. All quantified data are presented as mean ± SD

 We also noted that DM influenced the murine sperm parameters in terms of count abnormality and motility (Figure 5C). A significant increase in the number of sperms, higher rates of abnormality, and the lowest percentage of motility were indicated under diabetic conditions (*P* < 0.05; Figure 5C). Interestingly, it was noted that inhibition of autophagy in diabetic mice (DM+HCQ) resulted in a decline in the number of sperm cells compared to the matched control DM group (*P* < 0.01) and closed it to the basal control levels. In DM + Met, and DM + HCQ groups, the percentage of sperms with normal morphology and motility increased (*P* < 0.001; Figure 5C). Interestingly, Met and HCQ administration increased sperm counts and raised the percentage of abnormal sperm in non-diabetic conditions (*P* < 0.05). We found a non-significant difference in terms of sperm motility in DM mice after receiving Met and/or HCQ (*P* > 0.05; Figure 5C). These data support the notion that autophagy modulation, either stimulation or inhibition, can alter some sperm parameters in diabetic mice.

## Discussion

 This study was carried out to investigate the effect of autophagy on dynamic growth and regenerative potential of rat testicular organoids within the Alg/Gel hydrogel in response to autophagy stimulation/inhibition. How and by which mechanism autophagy modulation can affect the process of spermatogenesis was also monitored in diabetic mice.

 Data confirmed suitable physical and chemical properties indicated the successful ionic cross-linking between the Alg and Gel. For example, the degradation of Alg/Gel hydrogel initiated significantly after 4 days and reached a relatively steady state. Inside the body, the replacement of calcium ions with sodium ions can lead to the absorption of water molecules and the loosening of the polymeric network.^34^ With the initiation of degradation, the possibility of natural ECM production increases after being transplanted into the target sites.^35,36^ Having an interconnected porous microenvironment with an average pore size of 36.28 ± 13.61 µm, the migration of transplanted cells, and/or host tissue is facilitated into the deeper layer of hydrogel.^37^ This feature allows suitable penetration of nutrients and drainage of cell byproducts while immune cells can recruit the Alg-based grafts, leading to replacement with natural ECM at appropriate times.^38^ MTT data indicated an enhanced survival rate in cultured whole testicular tissue cells compared to the cells cultured on plastic surfaces. It was suggested the existence of 3D polymeric networks with several motifs in the structure Gel, a denatured collagen form, improves cultured cell attachment and phenotype acquisition.^27,39^ According to these data, natural hydrogels composed of Alg plus Gel are suitable for the expansion of different testicular tissue cells.

 It was noted that the addition of 5 µM Met, and 15 µM HCQ can efficiently stimulate, and inhibit the autophagic response in testicular organoids encapsulated within the Alg/Gel hydrogel. It has been indicated that Met can boost the autophagic response by increasing protein levels of Beclin-1, LC3-II/-I ratio, and reduction of P62 in different tissues such as the liver.^40^ The increase in total LC3 contents along with the conversion into LC3-II indicated lipidation and an increase of autophagosome formation. In later steps, these autophagosomes are directed toward lysosomes to form autophagolysosomes which further recycle the biomolecules or excrete them out of host cells.^41^ We also found that HCQ, an autophagy inhibitor, exerts opposite effects on testicular organoids after 7 days. The increase of P62 in the presence of HCQ indicated blockade of autophagic flux coincided with the accumulation of autophagosomes, and an incomplete autophagy response.^42^ It has been thought that excessive intracellular autophagolysosomes can contribute to a delay in the secretion of misfolded proteins and damaged organelles, making cells sensitive to different insulting conditions.^43^ These features indicate the success of the current protocol in the stimulation and inhibition of autophagy response inside the testicular organoids. The significant increase of SOD, and slight changes in TAC, and GPx activity in Met-treated organoids would be related to the fact that activation of autophagy can scavenge harmful free radicals during the culture of testicular organoids.^44^ Even though, the inhibition of autophagy using HCQ did not alter the activity of anti-oxidant enzymes related to the control conditions. As a common belief, HCQ can increase intracellular contents of reactive free radicals by the inhibition of autophagy response.^45^ However, the dose of HCQ used in current protocol was not at a level that causes oxidative stress as compared to the control levels.

 Emerging data have confirmed the putative effects of autophagy in the regulation of various cell bioactivities and differentiation potential under different biological conditions.^46,47^ We noted that Met and HCQ change key autophagy markers, but our data doesn’t show how that happens inside the cell. The most logical place to look next is the central signaling hub that controls autophagy. It’s well known that metformin can switch on AMPK. One of AMPK’s main jobs is to turn off mTOR, which acts as a major stop signal for autophagy. With mTOR off, the green light is given to ULK1 to start building autophagosomes.^48^ We think this AMPK-mTOR-ULK1 chain reaction is probably how metformin works in our organoids. HCQ, on the other hand, doesn’t stop the start of autophagy—it blocks the very end. It makes lysosomes less acidic so they can’t properly fuse with and digest the autophagosomes, causing everything to back up. Pinpointing this sequence—whether with phospho-antibodies or direct flux tests—is the critical missing link between our treatments and the cellular changes we observed.

 Histological examination indicated that treatment with autophagy activator (Met), and inhibitor (HCQ) led to the reduction of OCT3/4^+^ progenitors spermatogenic tubes of cultured organoids after 7 days. Despite the reduction of OCT3/4^+^ progenitors in Met, the number of maturing sperm cells such as spermatids, and spermatocytes was also reduced, indicating a diminishing cell population inside the seminiferous tubules. Considering the differences in the mode of action of HCQ, and Met, it seems that the reduction of OCT3/4^+^ progenitors, and maturing sperm cells can be promoted by engaging distinct effectors in both groups. For example, it was shown that in response to HCQ treatment, it was shown that the number of degenerated spermatocytes, and spermatogenic cell sloughing increased simultaneously with the cell cycle arrest and interstitial edema of rat testes.^49^ These features can promote the sloughing of all sperm cells and degenerative changes in spermatogonial stem cells as well. On the contrary, emerging data have proven the stimulatory effects of Met on sperm parameters such as motility, lactate production, acrosome reaction, etc.^50^ It seems that the reduction of OCT3/4^+^ progenitors is possibly related to the accelerated differentiation of these cells to maturing sperm cells.^51^ The significant activation of SOD in Met-treated organoids indicates the increase of basal reactive oxygen species and active mitochondrial function.^52^ The promotion of mitochondrial function and mitophagy in the presence of Met can expedite the differentiation of OCT3/4^+^ progenitors into the maturing sperm cells. Besides, the cytoskeletal remodeling is stimulated when the degradation of PDLIM1 is increased via autophagolysosomes under autophagic response, leading to the differentiation of spermatids.^53^

 It was found that excessive glucose levels can reduce sperm counts coincided with the increase of abnormal sperm features. This phenomenon is mainly due to the direct toxic effects of hyperglycemia on germ cells and the elimination of germinal layer seminiferous tubules.^54^ Besides, the reduction of steroidogenesis and the reduction of testicular matrix supporting cells such as Leydig cells can exacerbate DM-related pathologies.^55,56^ Like previously published reports, we indicated that the administration of Met in diabetic mice can restore sperm number, and motility while reducing the abnormal sperm counts.^57^ Met did not influence the number of progenitor cells within the seminiferous tubules. Also, the reduction of spermatogonial cells in the met and HCQ groups is completely consistent with the results of the previous stages and indicates the progression of cells towards mature cells. It seems that the therapeutic effects of Met on diabetic mice spermatogenesis can be related to different mechanisms. For example, it was shown that Met can improve testicular steroidogenesis and spermatogenesis via the expression of luteinizing hormone/chorionic gonadotropin receptors in diabetic rats.^58^ It should not be forgotten that adaptive autophagy response is inhibited in the testicular tissue of diabetic rodents, leading to reticulum endoplasmic injury.^59^ Thus, the stimulation of autophagy to normal levels can be an efficient strategy in diabetic conditions. Of course, caution should be taken that overactivation of autophagy not only does not restore spermatogenesis properties but also can exacerbate impaired spermatogenesis. For instance, it was indicated that the excessive autophagy response in high-fed diet mice diminished spermatogenesis potential and administration of chloroquine can blunt decreased fertility in obese, subfertile male mice.^60^ These data indicate the dual activity of autophagy in terms of spermatogenesis potential. Notably, the autophagic response is usually impaired or aborted under prolonged hyperglycemic conditions.^61^ Thus, activation of autophagy signaling pathway can be likely efficient in the restoration of spermatogenesis.^62^ Several aspects of our experimental design point to necessary future work. We used single doses and a single time point, so defining optimal treatment windows through detailed time-course and dose-response studies is essential. Our reliance on drug effects also means we lack genetic proof; using knockout models or lineage tracing would solidify the link between autophagy and specific germ cell fates. Furthermore, we inferred autophagic activity from protein markers. Direct flux measurements in vivo, alongside analysis of key hormones like testosterone and LH, would more firmly connect our treatments to testicular function. Finally, the unexpected increase in abnormal sperm in normal mice treated with either drug hints at complex effects on a balanced system, meriting a closer look in future studies. Addressing these points will be crucial to move from these promising morphological observations to a precise molecular and physiological understanding.

## Conclusion

 The current study aimed to study the possible effects of autophagy response on spermatogenesis capacity in rat testicular organoids incorporated into Alg/Gel hydrogel and diabetic mice. It was shown that autophagy modulation, either inhibition or stimulation, can alter spermatogenesis potential in terms of progenitor cell density, sperm counts, and parameters. Likewise, autophagy modulation in diabetic mice using Met had profound impacts on some sperm parameters and closed them to the control levels. Data from the present study indicate that the activation of adaptive autophagy response under diabetic conditions can in part but not completely restore testicular tissue function. Whether the inhibition and/or stimulation of autophagic response can exert therapeutic outcomes needs further investigation, as autophagy can act as dual swords in terms of cell biology. Taken together, under controlled conditions autophagy stimulation can be possibly a strategic medication in diabetic individuals.

## Competing Interests

 The authors declare no competing interests.

## Data Availability Statement

 All data that support our findings in this study are available from the corresponding authors upon reasonable demand.

## Ethical Approval

 This paper was published as partial fulfillment of the MSc student project “Investigating the effects of alginate hydrogels on differentiation capacity of spermatogonia stem cells through the autophagy Process” which was reviewed and approved on June 15, 2022, by the Research Ethics Committees of Laboratory Animals - Tabriz University of Medical Sciences (ethical code: IR.TBZMED.REC.1401.280).
